# Appendicitis Secondary to Obstructing Ascending Colon Malignancy

**DOI:** 10.7759/cureus.30165

**Published:** 2022-10-11

**Authors:** Jasminder K Malhi, Aleeha Noon, Jason J Londeree

**Affiliations:** 1 Internal Medicine, California Northstate University College of Medicine, Elk Grove, USA; 2 Plastic Surgery, California Northstate University College of Medicine, Elk Grove, USA; 3 General Surgery, Kaiser Permanente Sacramento, Sacramento, USA

**Keywords:** ascending colon, perforation, hemicolectomy, colon cancer, appendicitis

## Abstract

Appendicitis classically presents in a young adult as periumbilical pain localizing to the right lower quadrant as a result of an obstruction of the appendiceal orifice from lymphoid hyperplasia, fecalith, or infection. Persistent obstruction predisposes the appendix to an increasing inflammation, which may manifest as peritoneal signs as the disease course progresses toward perforation. Rarely, this obstruction occurs secondary to neoplastic growth, such as colonic adenocarcinoma. Furthermore, in older patient populations, appendicitis may not present with strict right lower quadrant pain. In this case report, we discuss an atypical presentation of perforated appendicitis with an underlying etiology of colonic adenocarcinoma in a 68-year-old male.

## Introduction

Acute appendicitis is a frequently occurring surgical emergency typically presenting with periumbilical abdominal pain that localizes to the right lower quadrant and more commonly arises in the second or third decade of one’s life [1-3]. The etiology commonly involves obstruction of the appendiceal orifice. Obstruction may occur secondary to etiologies such as lymphoid hyperplasia, fecaliths, infections, neoplasia, fibrosis, and calculi [4]. We present a case of appendicitis occurring due to one of the lesser common etiologies. The following case describes a patient presenting with appendicitis complicated by perforation and periappendiceal abscess formation in the setting of obstructive metastatic cecal adenocarcinoma. Moreover, this particular patient’s evolution of abdominal pain differs from the classic presentation. 

## Case presentation

The patient is a 68-year-old man with a past medical history of type II diabetes mellitus, hyperlipidemia, ascending colon malignancy, aortic atherosclerosis, and erectile dysfunction who presented to the emergency department with a chief complaint of bilateral lower quadrant abdominal pain progressively increasing in severity since the onset of one month ago. The location of pain remained confined to the bilateral lower quadrants over the course of the month. He also noted the pain acutely worsening over the two hours before seeking evaluation. His initial review of systems was also positive for nausea, and chills, as well as smaller and less frequent stools with his last bowel movement occurring two days ago. The patient was continuing to pass flatus. He denied fever and vomiting. 

**Figure 1:**
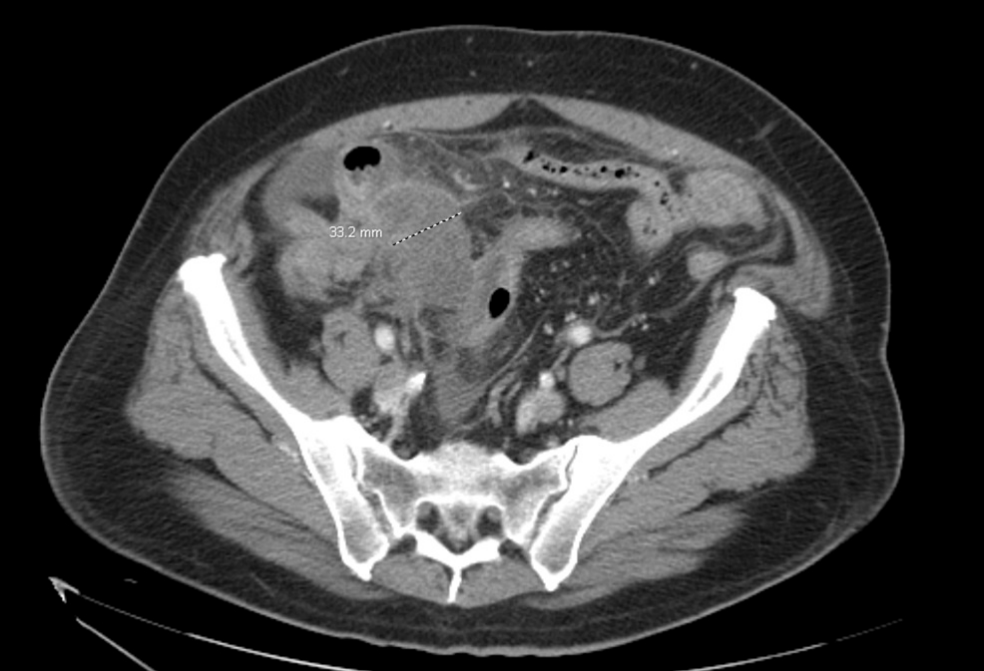
Marked dilated appendix arising from apex of cecum

**Figure 2:**
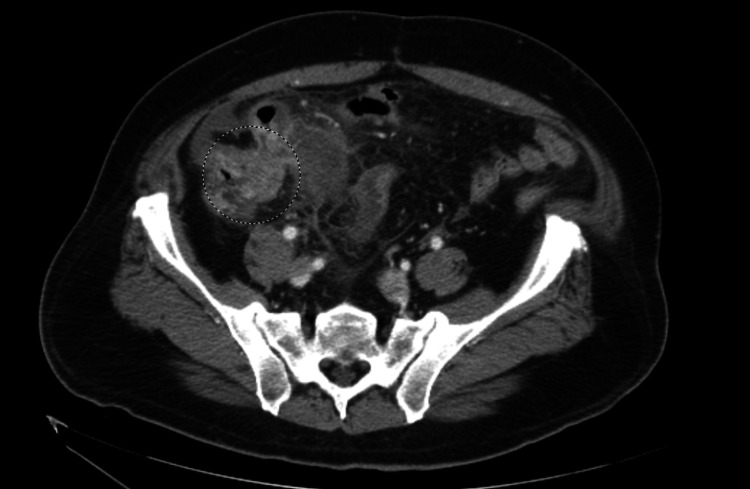
Known cecal mass with thickened irregular wall encircled

**Figure 3:**
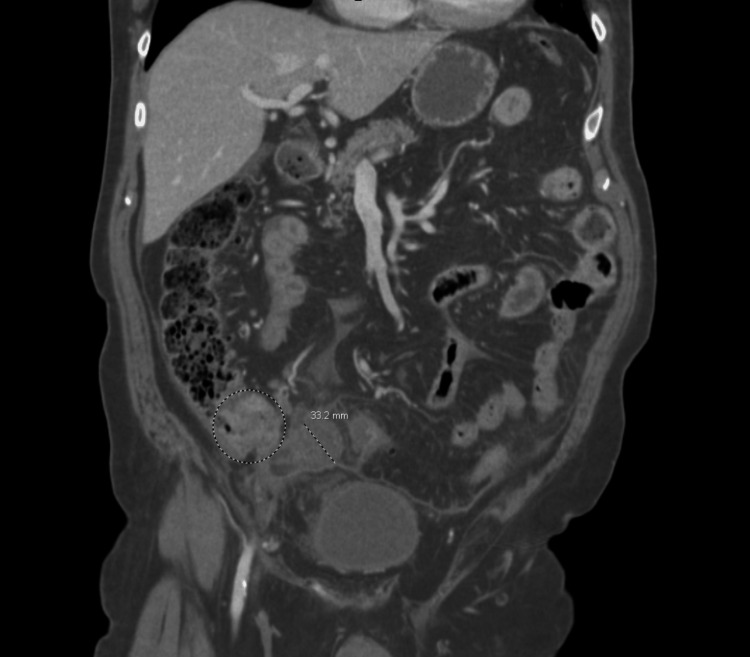
Coronal view showing close proximity of cecal mass and dilated appendix

Significant physical examination findings included left and right lower quadrant tenderness and voluntary guarding. The abdomen was non-distended. The remainder of the full examination was unremarkable. He was hemodynamically stable and afebrile. Initial laboratory studies were significant for lactate of 4.7 mmol/L with a repeat of 2.8 mmol/L, hyponatremia of 132 mEq/L, and mildly elevated aspartate aminotransferase and alkaline phosphatase of 44 U/L and 123 U/L, respectively. Leukocyte count was unremarkable at 9.7 k/uL. Imaging was significant for a computed tomography (CT) scan of the abdomen and pelvis with intravenous contrast demonstrating appendiceal dilation with a diameter of 3.3 cm and significant surrounding inflammatory stranding suggestive of acute appendicitis (Figure 1), minimal right lower quadrant and pelvic free fluid, and a previously identified cecal mass (Figure 2) and heterogenous right liver lobe mass suggestive of metastatic disease. In Figure 3, the cecal mass is shown in close proximity to the dilated appendix. The cecal mass was identified on colonoscopy and CT of the abdomen and pelvis two-and-a-half weeks prior to presentation. On the initial CT scan, the cecal mass was visualized near the ileocecal valve, obstructing the appendix causing appendiceal dilation with fluid and there was the collapse of the distal colon. A positron emission tomography (PET) scan confirmed hypermetabolism in the cecal mass, terminal ileum, and local lymph nodes, as well as a metastatic focus in the right liver lobe. 

The patient was admitted to surgery with suspected appendicitis, kept nothing by mouth, started on intravenous piperacillin-tazobactam, and monitored with serial abdominal evaluations. On hospital day 2, the patient conveyed improvement in pain and denied any bowel movements or passing flatus. Physical examination was similar to the initial presentation, but with decreased tenderness now more concentrated diffusely in the right lower quadrant and less so in the left lower quadrant. The patient was given laxatives resulting in multiple stools, in preparation for surgical intervention. Repeated laboratory results demonstrated decreasing lactate at 2.4 mmol/L and leukocytosis at 13.5 k/uL. 

On hospital day 3, the patient reported a further decrease in pain and the examination was significant for mild abdominal fullness and mild tenderness localized to the right lower quadrant. Laboratory results were significant for increasing leukocytosis of 14.5 k/uL. Blood cultures collected on admission were negative. The patient was taken to the operating room for laparoscopic right hemicolectomy with anastomosis and laparoscopic liver resection. During surgical intervention, small and large bowel distention was visualized along with a large cecal mass with adhesions, perforated appendicitis, purulent material in the abdominal cavity, and a hepatic mass measuring approximately 2 cm by 1.5 cm. Surgery was completed without any complications. Postoperatively, the patient continued to progress as expected with adequate multimodal pain control and appropriate abdominal examinations, increasing ambulation with a walker, appropriate incision appearance, and tolerating gradually advancing diet with the return of bowel function. The patient completed a five-day course of postoperative piperacillin-tazobactam. Laboratory results demonstrated a downtrending leukocyte count to 11.2 k/uL on postoperative day 2. Pathology results from specimens collected during surgery confirmed a 5 cm by 4 cm invasive moderately differentiated adenocarcinoma of the colon adjacent to the ileocecal valve and appendiceal orifice with negative margins and with the invasion of muscularis propria and involvement of pericolic fat with 2.5 cm hepatic metastatic focus; distal ileum showed no evidence of malignancy, but serositis was present. Additionally, two of the 25 lymph nodes were positive for metastatic disease. The malignancy was grade 2 and stage pT3N1bM1a. Pathology reports confirmed perforated appendicitis with periappendiceal abscess. The patient was discharged to home on postoperative day 3. The patient is currently undergoing a postoperative chemotherapy regimen consisting of oxaliplatin, leucovorin, and fluorouracil. The therapy regimen consists of one cycle of 28 days followed by 11 cycles of 14 days each. 

## Discussion

Appendicitis is classically described to present with periumbilical visceral pain, which then localizes to the right lower quadrant as the parietal peritoneum becomes inflamed. The pathophysiologic process of appendicitis stems from luminal obstruction, which is more commonly due to fecaliths or lymphoid hyperplasia secondary to infection in younger patients. Conversely, the etiology of obstruction in older individuals may sometimes be neoplastic processes, as demonstrated in this patient’s case [4]. 

This case interestingly highlights the presentation of appendicitis secondary to one of the lesser common etiologies, malignancy. Additionally, certain features of this patient’s presentation differ from the classical description. Rather than presenting initially with periumbilical pain, this patient presented with diffuse lower abdominal pain, which later localized to the right lower quadrant. Moreover, this patient’s appendix had perforated, as evidenced by the right lower quadrant and pelvic free fluid on imaging with purulent material visualized in the abdominal cavity during the surgical intervention and periappendiceal abscess formation. However, the patient remained afebrile with a relatively mild abdominal examination. Additionally, this patient experienced a more prolonged and indolent progression of symptoms which can be explained by the etiology being colon malignancy with the complication of appendiceal abscess formation [5]. The presence of fever and higher leukocytosis would have been even more suggestive of perforation [1]. 

It is also important to discuss that the presentation of appendicitis in patients of older age may be atypical regardless of etiology. In the case of the patient presented in this report, the etiology of his diagnosis was clear given his history of colon cancer. However, elderly patients may often present with less acute complaints of abdominal pain in a non-classical distribution. Despite this, signs of peritonitis are nearly always present on examination in this population [6]. 

## Conclusions

Appendicitis typically has the presentation of periumbilical to right lower quadrant pain, with additional symptoms such as fever and a higher degree of leukocytosis suggesting progression to perforation. In some instances, a perforated appendix may be a result of progressive colonic adenocarcinoma. When appendicitis presents atypically, both in symptoms and patient population (i.e. diffuse lower abdominal pain in an older male), it is important to consider underlying etiologies beyond a more common source such as a fecalith. By taking into consideration these less common etiologies, the patient’s treatment plan can be better tailored to optimize their health outcomes, as in this patient.
